# Author response to: Mesenteric SParIng *versus* extensive mesentereCtomY in primary ileocolic resection for ileocaecal Crohn’s disease (SPICY): study protocol for randomized controlled trial

**DOI:** 10.1093/bjsopen/zrac081

**Published:** 2022-06-03

**Authors:** Eline M.L. Van der Does de Willebois, Christianne J. Buskens

**Affiliations:** Department of Surgery, Amsterdam UMC, Location AMC, Amsterdam, The Netherlands

*Dear Editor*

Thank you for your letter in response to our study protocol^1^. We agree that both the extent of mesenteric resection and the role of anastomotic technique are important factors to consider when aiming to reduce postresectional Crohn’s disease recurrence. However, we disagree about your methodological proposal to analyse both research questions in one design.

Current surgical practice for Crohn’s disease resection includes several anastomotic configurations (end-to-end, side-to-side, Kono-S), but there are also different anastomotic techniques, which might impact on surgical efficacy. It has been suggested that an end-to-end (ETE) anastomosis might be superior to a side-to-side (STS) as it could reduce stasis thereby reducing recurrence and improving gastrointestinal function. Gajendran *et al*.^2^ demonstrated that patients with a surgical reconstruction of the bowel as an intact tube (ETE) had improved quality of life and less healthcare utilization when compared with STS reconstruction. In addition to the configuration, there might be a difference in wound healing between these two techniques. The inverted stapled anastomosis without direct mucosa–mucosa alignment is fundamentally different from a handsewn anastomosis. The STS is associated with ulcerations at the staple line, which may not have healed after 6 months when endoscopic surveillance in Crohn’s patients is advised according to current guidelines, leading to systematic over scoring of Crohn’s recurrence. The Kono-S is a different type of side-to-side anastomosis with the additional factors of being handsewn and exclusion of the mesentery.

We believe that there are too many factors/treatment arms to reliably analyse in one study design and would necessitate a further standard handsewn ETE anastomosis arm.

As discussed in the study protocol, we feel that the optimal type of anastomosis is a different research question from our aim to analyse the impact of mesenteric resection in reducing postoperative Crohn’s recurrence.

*Disclosure*. The authors declare no conflict of interest.
